# Risk Factors for Posttraumatic Stress Disorder Among Deployed US Male Marines

**DOI:** 10.1186/1471-244X-10-52

**Published:** 2010-06-25

**Authors:** Christopher J Phillips, Cynthia A LeardMann, Gia R Gumbs, Besa Smith

**Affiliations:** 1 Department of Defense Center for Deployment Health Research, Naval Health Research Center, Department 164, 140 Sylvester Road, San Diego, CA 92106, USA

## Abstract

**Background:**

Combat exposure has been reported as one of the strongest risk factors for postdeployment posttraumatic stress disorder (PTSD) among military service members. Determining the impact of specific deployment-related exposures on the risk of developing PTSD has not been fully explored. Our study objective was to explore the relationship between specific combat exposures and other life experiences with postdeployment PTSD.

**Methods:**

This study consisted of male Marines who completed a Recruit Assessment Program (RAP) survey during recruit training at the Marine Corps Recruit Depot in San Diego, California as well as a follow-up survey several years after recruit training. Study participants included those Marines who deployed to the current operations in Iraq or Afghanistan between the baseline and follow-up surveys. Multivariable logistic regression was performed to determine which significant exposures and experiences were associated with postdeployment PTSD.

**Results:**

Of the 706 study participants, 10.8% screened positive for postdeployment PTSD. Those who reported feeling in great danger of death (odds ratio [OR] = 4.63, 95% confidence interval [CI]: 2.46-8.73), were shot or seriously injured (OR = 3.51, 95% CI: 1.58-7.77), saw someone wounded or killed (OR = 2.47, 95% CI: 1.08-5.67), and baseline (before recruit training) prior violence exposures (OR = 2.99, 95% CI: 1.46-6.10) were at increased odds for reporting PTSD symptoms. Number of deployments, number of close friends or relatives reported at follow-up, and enlisted pay grade were also significantly associated with postdeployment PTSD.

**Conclusions:**

Combat exposures, specifically the threat of death, serious injury, and witnessing injury or death are significant risk factors for screening positive for postdeployment PTSD among male Marines as well as violence exposures prior to entering the Marine Corps, which are independent of future combat exposures. A thorough history of lifetime violence exposures should be pursued when considering a clinical diagnosis of PTSD.

## Background

Posttraumatic stress disorder (PTSD) may develop after an individual witnesses or experiences a traumatic event, such as a natural disaster, combat, or violent personal assault [1]. While not everyone who experiences traumatic events will develop PTSD, factors including the intensity of the trauma and proximity to the event can elevate one's propensity for developing the disorder [2-4]. Unrelated to the traumatic event, additional risk factors for developing PTSD include younger age at the time of the trauma, lower social economic status, family history of psychiatric illness, prior assault, childhood adversities, female gender, minority race, and lack of social support [3,5-14]. PTSD symptoms following deployment to Iraq or Afghanistan have been associated with lower rank, being unmarried, less formal education, and a history of childhood adversity [14,15].

Among military service members, combat exposures are reported as the strongest predictors of subsequent PTSD [6-8,11,14,16-20]. In several studies of war veterans, increased rates of PTSD were demonstrated among those sustaining wounds, those deployed as part of ground units, and those discharging their weapon or witnessing persons being wounded or killed [4,18,21]. Other studies, however, have demonstrated that the most important war-zone factor is not actual combat exposure, but the perceived threat of personal danger [15,20,22].

Irrespective of combat exposure, a recent cross-sectional study of soldiers and Marines several months after returning from Iraq found approximately 7.6% with probable new onset PTSD [21]. Further, 8.5% of deployed Marines screened positive for new onset PTSD symptoms in a prospective cohort study following deployment in support of the operations in Iraq or Afghanistan [14]. Determining the influence of specific deployment-related exposures and military experiences on the risk of developing PTSD requires further research.

The Recruit Assessment Program (RAP) study, conducted at the Marine Corps Recruit Depot (MCRD), San Diego, California, collects comprehensive baseline health data from Marine recruits in the first days of the 12-week recruit training [23,24]. Several years after the completion of the RAP survey, the US Marine Corps Health Assessment Project (RAP II) resurveyed a portion of RAP responders who successfully completed boot camp. This follow-up survey evaluated health status as well as obtained deployment and exposure information. Using follow-up exposure data from RAP II, associations between exposures and postdeployment mental health outcomes were examined. Rarely have young Marines early in their military career been available for detailed study with the ascertainment of preservice and predeployment risk factors and postdeployment exposures. This study is one of the first to prospectively assess the impact of specific deployment-related exposures, other potentially related military experiences, and prior violence exposures with the risk of postdeployment PTSD among Marines.

## Methods

### Study Population

Since June 2001, the RAP study has collected baseline health data on Marine recruits at MCRD San Diego. The RAP survey was developed through a collaborative effort involving public health officials, clinicians, and researchers from the Department of Defense, Veterans Health Administration, and Department of Health and Human Services. The survey instrument includes questions on demographics, health, family history, prior violence exposures, tobacco and alcohol use, and psychological history. The RAP II survey was conducted from 2004 until 2006 as a follow-up mailed survey of Marines who completed a RAP questionnaire between October 2001 and October 2002 and successfully completed boot camp. This follow-up survey included many of the same questions from the initial baseline survey, but also included questions on mental health, deployment history, and combat exposures. Of the 19,089 individuals eligible to receive a follow-up survey, 11,640 had a reliable mailing address obtained from the Defense Manpower Data Center (DMDC). Survey mailings followed standard Dillman procedures, beginning with an introductory postcard, followed by a cover letter and survey (survey packet), then a reminder postcard, and up to two survey packets and reminder postcard re-mailings [25].

The population for this current study included RAP II responders who gave voluntary informed consent, had been deployed for at least 30 days in support of Operation Enduring Freedom or Operation Iraqi Freedom (OEF/OIF) between the baseline and follow-up, and had complete demographic, exposure, and behavioral data. Since MCRD San Diego does not train female recruits, analysis in this study was restricted to males. This study was conducted with prior approval by the Naval Health Research Center's Institutional Review Board.

### Deployment Data

Deployment data, obtained from DMDC, included all service members deployed in support of OEF/OIF from 2001 onwards. These data were used to determine number of deployments, length of last deployment, cumulative length of deployment, gap time, and deployment location. The number of in-theater days during the most recent deployment prior to completion of the follow-up survey was used to determine the length of last deployment. Cumulative length of deployment was calculated as the total number of days deployed for all deployments that occurred between completion of the baseline and follow-up surveys. Gap time was calculated as the number of days between the last deployment and completion of the follow-up survey. Responders who completed the follow-up survey while deployed were assigned a gap time of zero days.

### Posttraumatic Stress Disorder Assessment

The PTSD Checklist-Civilian Version (PCL-C), a 17-item self-report measure of PTSD symptoms included in the follow-up survey, was used to assess PTSD [26,27]. Using a 5-point Likert scale from 1 (*not at all*) to 5 (*extremely*), responders rated the severity of intrusion, avoidance, and hyperarousal symptoms during the past 30 days. In this study, participants met the criteria for potentially clinically relevant PTSD symptoms if they had a total score of 50 or more on a scale of 17 to 85 points, in addition to meeting the *Diagnostic and Statistical Manual of Mental Disorders*, 4th Edition (DSM-IV) criteria [1,26-30]. In order to meet the DSM-IV criteria, participants had to report a moderate or above level of at least one intrusion symptom, three avoidance symptoms, and two hyperarousal symptoms [1]. Using this instrument with a cut point of 50 is less likely to generate false positives and has been reported to be highly specific (specificity, 99%), while less sensitive (60%) [30].

### Exposures of Interest

Using questions from the follow-up survey, six individual combat exposures were assessed. Five of the combat exposures were based on affirmative responses to the following questions, in regard to deployment: (1) "Were you engaged in direct combat where you discharged your weapon?", (2) "Were you ever shot or seriously injured?", (3) "Did you personally see anyone wounded, killed, or, dead?", (4) "Do you think you were exposed to any chemical, biological, or radiological warfare agents?", (5) "Did you enter or closely inspect any destroyed military vehicles?" Furthermore, responders who answered, "*often*" or "*very often*" to the question, "How often did you feel that you were in great danger of being killed?" were positively assessed with this combat exposure. Finally, a combat exposure score between 0 and 6 was created based on one point for an affirmative response to each form of exposure.

### Covariate Information

Age, race/ethnicity, education level, adverse childhood experiences, prior trauma history, number of close friends or relatives, and potential problem drinking were assessed at baseline. Adverse childhood experiences occurring before the age of 17 years old were assessed using seven questions from the Adverse Childhood Experiences Study [31,32], the Childhood Trauma Questionnaire [33,34], and the Conflict Tactics Scales [35]. These domains included physical neglect, physical abuse, emotional neglect, emotional abuse, domestic violence, childhood sexual abuse and exposure to household substance abusers. An adverse childhood experience score between 0 and 7 was created from the sum of affirmative responses. To assess number of close friends or relatives, responders were asked, "How many close friends or relatives do you have that you can call on for help or talk to about personal problems?" A summary score (0-7) for exposure to violence before entering Marine recruit training was based on affirmative responses to the following events: (1) being in an accident where they could have been killed but were not badly hurt, (2) being in an accident where they were injured and had to spend at least one night in the hospital, (3) seeing a close family member or friend being badly injured or killed, (4) seeing a stranger being badly injured or killed, (5) being seriously attacked, beaten up, or assaulted, (6) being threatened with a knife, gun, club, or other weapon, and (7) being raped. Regarding alcohol, responders who reported feeling at least one of the following during the year prior to completing the baseline survey, (1) the need to cut down, (2) annoyed at someone who suggested they cut down on drinking, (3) guilty after drinking, or (4) needing a drink first thing in the morning, were defined as having potential problem drinking. Questions from the followup survey were used to assess pay grade and reassess the number of close friends or relatives. Pay grade was categorized as junior enlisted (E1-E3) and noncommissioned officer (E4-E5) with noncommissioned officers having more responsibilities compared to junior enlisted. At followup, number of close friends was assessed using the same baseline question. When possible, DMDC data supplemented missing demographic data for age, education, and race/ethnicity.

### Statistical Analysis

Univariate analyses were performed to compare age, education level, race/ethnicity, adverse childhood experiences, exposure to prior violence, potential problem drinking, and general health between the deployed, nonresponders of RAP II and the study sample as potential variables for non-response bias weighting. Age, education and number of deployments were the only characteristics that were significantly different between responders and nonresponders, and used to develop sample-based weights which were included in the multivariable model to adjust for any potential non-response bias. Descriptive and univariate analyses were completed to compare deployment exposures between responders with PTSD symptoms and those without PTSD symptoms. Manual backward stepwise multivariable logistic regression was performed to investigate the association between deployment exposures and PTSD symptoms. The saturated model included age, education, race/ethnicity, enlisted pay grade, adverse childhood experiences, prior exposure to violence, potential problem drinking, number of close friends (baseline), number of close friends (follow-up), number of deployments, gap time, total days deployed, number of combat exposure types, and six deployment exposures: discharging a weapon, shot/seriously injured, felt in great danger of being killed, personally witnessing someone wounded, killed, or dead, belief of exposure to chemical/biologic/radiologic weapons, inspecting destroyed military vehicles. A multiplicative interaction term, number of friends at follow-up and number of combat exposure types in relation to PTSD symptoms was tested in the saturated model and was not significant. Length of last deployment was not included in the saturated model due to its high variance inflation factor (multicollinearity). Nonsignificant variables were manually removed one at a time from the multivariable model if they did not confound the relationship between "felt in great danger of being killed" and PTSD by more than 15%. The final multivariable model was adjusted for the remaining deployment exposures and included only those covariates that were significantly associated with the outcome. Regression diagnostics, including examining covariates for multicollinearity and model fit by R^2^, were performed. All data analyses were completed using SAS (version 9.2 SAS Institute, Inc., Cary, North Carolina).

## Results

Of the 11,640 RAP participants who were sent a follow-up survey, 8,354 (71.8%) had deployed at least 30 days. Of those deployers 1,114 (13.3%) returned a survey. Among the responders, those who did not consent (*n = *51) or completed the follow-up survey before their first deployment started (*n = *262), were excluded from this study. Responders who did not complete the PTSD screening questions (*n = *18) or had other missing responses to covariate or exposure data (*n = *77) were also excluded. Of the 706 Marines in the study population, the majority were younger than 21 years old, high school educated or less, and White non-Hispanic (Table 1). Nearly half (44%) of the responders accumulated between 121 and 240 days total deployment time. The most common length of their last deployment before completing the follow-up survey was 121 to 240 days (62%, *n = *441) and the median gap time was 76 days.

**Table 1:** Demographic and Deployment Characteristics of US Marine Corps Health Assessment Project Participants

	Study population *N *= 706	
	*n*	**%**^**a**^
**Baseline characteristics**		
Age, years		
17-20	553	78.3
21-31	153	21.7
Education		
High school or less	528	74.8
More than high school	178	25.2
Race/ethnicity		
White non-Hispanic	474	67.1
Hispanic	166	23.5
Other	66	9.4
Adverse childhood experiences		
0	353	50.0
1	244	34.6
2-7	109	15.4
Prior violence exposures		
0	407	57.6
1	179	25.4
2-7	120	17.0
Potential problem drinking		
No	644	91.2
Yes	62	8.8
Number of close friends/relatives		
0-2	203	28.7
3-4	261	37.0
5 or more	242	34.3
**Follow-up characteristics**		
Number of close friends/relatives		
0-2	202	28.6
3-4	222	31.4
5 or more	282	39.9
Enlisted pay grade		
Junior enlisted (E1-E3)	334	47.3
Noncommissioned officer (E4-E5)	372	52.7
Number of deployments		
1	433	61.3
2	273	38.7
Length of last deployment		
1-120 days	211	29.9
121-240 days	441	62.5
>240 days	54	7.7
Cumulative length of deployments^b^		
31-120 days	136	19.3
121-240 days	308	43.6
241-360 days	172	24.4
>360 days	90	12.8
Gap time^c^		
0 days	257	36.4
1-180 days	222	31.4
>180 days	227	32.2
Number of Combat Exposures Types		
0-1	267	37.8
2-3	257	36.4
4-6	182	25.9

^a^Percents may not add to 100 due to rounding.

^b^Cumulative number of days deployed for all deployments that occurred between baseline and follow-up surveys.

^c^Number of days from last deployment to follow-up survey completion. If survey was completed while on deployment, gap time equals zero.

The overall prevalence of those who screened positive for PTSD was 10.8% at follow-up. The median PCL-C score for those with PTSD was 58.5, more than twice the median score of 26 for those without PTSD. Several distinct combat exposures significantly increased the risk for PTSD in the univariate analysis (Table 2), including being shot or seriously wounded; feeling in great danger of being killed; seeing someone wounded, killed, or dead; discharging a weapon; and entering or closely inspecting destroyed military vehicles. The number of combat exposure types was also positively associated with PTSD. In additional univariate analysis, adverse childhood experiences, prior violence exposures, pay grade, number of deployments, number of close friends or relatives at follow-up, and race/ethnicity were associated with PTSD (Table 2).

**Table 2:** Univariate Prevalence and Unadjusted Odds Ratios of PTSD Following Deployment among United States Marine Corps Health Assessment Project Participants, by Demographic and Exposure Characteristics

	Study population			**PTSD**^**a**^	
	N	n	%	**OR**^**b**^	**(95% CI)**^**b**^
**Overall**	**706**	**76**	**10.8**		

**Baseline characteristics**					
Age, years					
17-20	553	58	10.5	1.00	
21-31	153	18	11.8	1.14	(0.65-2.00)
Education					
High school or less	528	60	11.4	1.00	
More than high school	178	16	9.0	0.77	(0.43-1.38)
Race/ethnicity					
White non-Hispanic	474	44	9.3	1.00	
Hispanic	166	19	11.4	1.26	(0.72-2.23)
Other	66	13	19.7	**2.40**	**(1.21-4.74)**
Adverse Childhood Experiences					
0	353	33	9.3	1.00	
1	244	24	9.8	1.05	(0.61-1.84)
2-7	109	19	17.4	**2.05**	**(1.11-3.77)**
Prior Violence Exposures					
0	407	31	7.6	1.00	
1	179	24	13.4	**1.88**	**(1.07-3.30)**
2-7	120	21	17.5	**2.57**	**(1.42-4.67)**
Potential problem drinking					
No	62	6	9.7	1.00	
Yes	644	70	10.9	0.88	(0.37-2.11)
Number of close friends/relatives					
0-2	203	26	12.8	1.13	(0.64-1.98)
3-4	261	30	11.5	1.00	
5 or more	242	20	8.3	0.69	(0.38-1.26)
**Follow-up and deployment characteristics**					
Number of close friends/relatives					
0-2	202	41	20.3	**2.20**	**(1.27-3.82)**
3-4	222	23	10.4	1.00	
5 or more	282	12	4.3	**0.38**	**(0.19-0.79)**
Enlisted pay grade					
Junior enlisted (E1-E3)	334	48	14.4	**2.08**	**(1.26-3.33)**
Noncommissioned officer (E4-E5)	372	28	7.5	1.00	
Number of deployments					
1	433	34	7.9	1.00	
2	273	42	15.4	**2.13**	**(1.32-3.45)**
Length of last deployment					
1-120 days	211	29	13.7	1.00	
121-240 days	441	42	9.5	0.66	(0.40-1.09)
>240 days	54	5	9.3	0.64	(0.24-1.74)
Cumulative length of deployments^c^					
31-120 days	136	12	8.8	1.00	
121-240 days	308	29	9.4	1.07	(0.53-2.17)
241-360 days	172	23	13.4	1.60	(0.76-3.33)
>360 days	90	12	13.3	1.59	(0.68-3.72)
Gap time^d^					
0 days	257	28	10.9	1.00	
1-180 days	222	18	8.1	0.72	(0.39-1.34)
>180 days	257	30	13.2	1.25	(0.72-2.16)
Discharged a weapon					
No	386	21	5.4	1.00	
Yes	320	55	17.2	**3.61**	**(2.13-6.11)**
Shot or seriously injured					
No	665	61	9.2	1.00	
Yes	41	15	36.6	**5.71**	**(2.87-11.36)**
Felt in great danger of being killed					
Never/once/few times	399	17	4.3	1.00	
Often/very often	307	59	19.2	**5.35**	**(3.05-9.38)**
Personally saw someone wounded, killed, or dead					
No	248	9	3.6	1.00	
Yes	458	67	14.6	**4.55**	**(2.23-9.29)**
Believed you were exposed to any chemical, biological, or radiological warfare agents					
No/don't know	683	71	10.4	1.00	
Yes	23	5	21.7	2.39	(0.86-6.65)
Entered or closely inspected destroyed military vehicles					
No	320	21	6.6	1.00	
Yes	386	55	14.2	**2.37**	**(1.40-4.01)**
Number of Combat Exposures Types					
0	145	4	2.8	1.00	
1-3	379	29	7.7	**2.92**	**(1.01-8.48)**
4-6	182	43	2.4	**10.90**	**(3.80-31.3)**

*Note*. PTSD, posttraumatic stress disorder; OR, odds ratio; CI confidence interval; PCL-C, PTSD Patient Checklist-Civilian Version; DSM-IV, Diagnostic and Statistical Manual of Mental Disorders, 4^th ^ed.

^a^Posttraumatic stress disorder based on PTSD Patient Checklist-Civilian Version with *Diagnostic and Statistical Manual of Mental Disorders*, 4th Edition criteria and a sum of 50 points out of 85 points possible on the follow-up questionnaire.

^b^Unadjusted odds ratios and associated 95% confidence intervals.

^c^Cumulative number of days deployed for all deployments that occurred between baseline and follow-up surveys.

^d^Deployment occurred between baseline and follow-up in support of OEF/OIF operations.

In the reduced model, after removing variables that were neither significant nor confounders, Marines who reported feeling in great danger of being killed often or very often (odds ratio [OR] = 4.63, confidence interval [CI]: 2.46-8.73); were shot or seriously injured (OR = 3.51, CI = 1.58-7.77); witnessed someone being wounded, killed, or dead (OR = 2.47, CI = 1.08-5.67), and were deployed twice in support of OIF/OEF (OR = 1.91, CI = 1.10-3.33) compared with those who did not report these exposures were significantly more likely to screen positive for postdeployment PTSD (Table 3). Junior enlisted Marines compared to noncommissioned officers were at higher odds for PTSD (OR = 2.15, CI = 1.20-3.85), as were those Marines with 2 or more prior violence exposures (OR = 2.99, CI = 1.46-6.10). Marines who reported having 5 or more close friends or relatives were at decreased odds (OR = 0.26, CI = 0.12-0.59) for PTSD compared with Marines who reported having 3 to 4 close friends or relatives at follow-up (Table 3).

**Table 3:** Adjusted Odds of Posttraumatic Stress Disorder from a Reduced Multivariable Logistic Regression Model

	Study Population *N *= 706	
	OR	(95% CI)
**Baseline characteristic**		
Prior Violence Exposures		
0	1.00	
1	1.96	(0.99-3.85)
2-7	**2.99**	**(1.46-6.10)**
**Follow-up and deployment characteristics**		
Number of close friends/relatives you can confide in		
0-2	1.80	(0.96-3.36)
3-4	1.00	
5 or more	**0.26**	**(0.12-0.59)**
Enlisted pay grade		
Junior enlisted (E1-E3)	**2.15**	**(1.20-3.85)**
Noncommissioned officer (E4-E5)	1.00	
Number of deployments		
1	1.00	
2	**1.91**	**(1.10-3.33)**
Shot or seriously injured		
No	1.00	
Yes	**3.51**	**(1.58-7.77)**
Felt in great danger of being killed		
Never/once/few times	1.00	
Often/very often	**4.63**	**(2.46-8.73)**
Personally saw someone wounded, killed, or dead		
No	1.00	
Yes	**2.47**	**(1.08-5.67)**

Note. Odds ratios (OR) and associated 95% confidence intervals (CI) are adjusted for all other variables in the table.

^a^Posttraumatic stress disorder based on PTSD Patient Checklist-Civilian Version with *Diagnostic and Statistical Manual of Mental Disorders*, 4th Edition criteria and a sum of 50 points out of 85 points possible on the follow-up questionnaire. Model fit by R^2 ^= 0.851

## Discussion

The rates of mental health morbidity among soldiers and Marines returning from deployment in support of the wars in Iraq and Afghanistan may be as high as 20% [21]. Two separate investigations described proportions of PTSD between 12.2% and 12.9% in soldiers and Marines 3 to 4 months after combat exposure in OEF/OIF [21,36]. The current study of male Marines who deployed for at least 30 days found 10.8% screened positive for PTSD during or following deployment in support of OEF/OIF. Because of the high proportion of PTSD among soldiers and Marines, it is essential to determine factors that protect against or increase the risk for PTSD among these service members.

After adjustment in the regression model, feeling in great danger of death was the strongest independent predictor of PTSD, followed by being shot or seriously injured, prior violence exposures, and personally seeing someone wounded, killed, or dead. These findings are consistent and supportive of results from several earlier studies [6-8,11,13,14,16-21,37,38].

Using the same strict PCL criteria applied in the current study, Hoge [21] found that 12.2% of Marines experienced new onset or chronic PTSD symptoms. The slightly lower proportion of postdeployment PTSD symptoms in the current study (10.8%) may reflect slightly lower levels of combat exposures reported by these Marines. While it is difficult to make an exact comparison of exposure histories, since the two studies used different questions to assess combat experiences, the equivalent questions have higher affirmative responses among the population in the Hoge study. For each comparable dimension of combat exposure within the two studies, there appears to be a dose-response phenomenon; greater exposure increases the likelihood for PTSD symptoms.

Traumatic combat exposure alone [6,39] is not necessarily a sufficient factor for the development of PTSD. The probability is dependent upon the "range and variance" of traumatic exposure types [40]. Exposure to violence as a civilian, prior to the Marine Corps training, broadened the range and expanded the exposure types for the cohort subjects. As we describe, reporting between 2 to 7 violence episodes was strongly associated with postdeployment PTSD. These antecedent violence exposures may represent the "building block" effect [41], perhaps the consequence of a neural fear network, enlarged in response to new traumatic events and types [42].

Social support was assessed at baseline and follow-up by asking responders about the number of close friends or relatives they can call or ask for help when they have a problem. There was no association of baseline social support with screening positive for postdeployment PTSD. Interestingly, and consistent with previous research that describes the buffering effect of social support on PTSD symptoms [15,43-47], we found that reporting 0 to 2 close friends or relatives at follow-up was associated with a non significant increase in odds for PTSD, while reporting 5 or more close friends/relatives was associated with a significant reduction in odds for PTSD. Summarized literature suggests an interactive cycle between social support resources and PTSD in which either can influence the nature or expression of the other [48]. The disorder itself is defined by feelings of detachment or estrangement from others, with half of the six required symptoms for a positive PCL-C screen being expressions of avoidance. There is evidence that a strong social support network, indicated by unit cohesion, is protective [49], a large social support network may diminish the association between stressful life events and PTSD symptoms. Whether PTSD leads to social avoidance behaviors, or a large social support network with trusted friends and relatives who lessen the opportunity for detachment and estrangement reduces the likelihood of developing PTSD cannot be definitively determined from this study. Social support was measured at baseline and follow-up in this study, however the baseline assessment may not accurately reflect the number of close friends and relatives a Marine has immediately prior to deployment since the baseline survey was completed during the first days of Marine Corps training. The follow-up assessment of social support was ascertained at the same time as PTSD, so while we suspect the association with smaller social support networks measured at follow-up is a consequence of PTSD, we cannot be certain. Longitudinal studies that can better control for predeployment assessed levels of social support, during deployment, and in the immediate postdeployment period are crucial to understanding the influence of social support on symptom mediation [50-53].

Consistent with previous studies and independent of age, higher ranking noncommissioned officers (E4-E5) had decreased odds for PTSD compared with junior enlisted Marines (E1-E3) [15,36]. It is likely that the same qualities valued by Marines that are indicative of excellence amongst their ranks, such as mental stamina and competency, enhance their promotion potential, and may also increase resiliency to developing PTSD [45]. In this study, we were able to control for predeployment rank, since all responders were recruits at baseline, but future longitudinal studies could examine how quickly Marines are promoted to further investigate this relationship.

Marines with two deployments were at a significant increase in odds for a positive PTSD screen. Multiple, not solitary, deployments have become more common as the conflicts in Iraq and Afghanistan continue. It is reasonable to speculate that additional deployments increase the probability for exposure to additional or more traumatic events such as witnessing death, being shot or seriously wounded, or fearing one's own impending death. Indeed, this proved to be true for the study cohort. On average, those deployed once experienced 1.76 combat exposure types while Marines who deployed twice experienced 2.83 combat exposure types.

There are several limitations to this study that should be noted. Participants in the study were a self-selected cohort who consented to participate after invitation in the RAP II survey. The survey was not anonymous, which may have led, due to fear of stigma and reticence, an underreporting of sensitive topics, including adverse childhood symptoms and PTSD symptoms. This lack of anonymity may have been a factor in the low 13% response rate as well. While responders were slightly more likely to be younger and have more than a high school education, the current analysis compensated for these differences with sampling weights. Moreover, the prevalence of PTSD symptoms in this population may be incorrectly estimated secondary to the higher proportions of younger and more educated Marines than are typically represented in the enlisted US Marine Corps. While Marines who successfully complete basic training are assumed to be of good health, PTSD symptoms were not assessed before deployment, therefore no baseline burden was available to estimate new onset of postdeployment PTSD symptoms. We examined a summed score of the different combat exposures types; however, we could not assess the frequency of each exposure or measure the degree of distress for each exposure event. The same exposure could have been perceived as more or less distressing by different subjects, and vary within the same subject at different times. Another limitation of this study is that we did not have the available data to develop measurement scales that meet the standards of psychometric principles, nor were our deployment exposure variables based on an established instrument with proven psychometric properties. For example, we did not have (1) multiple waves of data to test for the reliability of our measures over time (test-retest reliability); (2) the benefit of a large number of measures tapping the same latent construct to develop scales that were internally consistent; nor (3) any data to validate our exposure measures against established instruments with proven psychometric properties. However, our use of single item exposure variables did reflect systematic variation across the study cohort and yielded meaningful results. Responders were all male Marines, so inferences from these data to female Marines and other service branches must be considered with caution. Although the PCL-C is a surrogate for a clinician's diagnosis and may misclassify PTSD status for some responders, it is a standardized instrument that has been validated in other populations [30,54]. Finally, exposures and some covariates were from self-reported data, which inherently have some recall and reporting biases.

Despite these limitations, this study had many strengths. Baseline and follow-up survey data as well as electronic military data were available on multiple metrics, which allowed for adequately addressing many potential confounders. Weighting was employed to reduce potential nonresponse bias. Additionally, the survey instruments contained many exposure questions which allowed the current study to uniquely address deployment experiences beyond whether they were in combat situations. Most research thus far has focused on predictors of PTSD that occurred during or after the traumatic exposure; the assessment of exposure to violence as civilians, prior to Marine Corps training, allowed us to account for the predeployment exposure to violence. PCL-C screening for PTSD may more accurately capture those with PTSD symptoms compared with ascertainment via ambulatory or hospitalization data, since many patients with symptoms may not seek treatment. Our survey captured many of the participants approximately 3 months after return from deployment and likely represents the optimal time window to ascertain PTSD symptoms [21,55].

## Conclusions

This study adds to the growing body of literature for the OEF/OIF conflicts that demonstrates that combat exposures, specifically the threat of death, serious injury, and/or witnessing injury or the death of others are the most significant traumatic risk factors associated with PTSD among male Marines. Our findings reveal an additional risk factor, exposure to violence before entrance into the Marine Corps, which is independent of future combat exposures. We are unaware of any recent studies with young Marines, at the earliest phases of the military experience, able to examine the relationship among prior violence exposures as young adults prior to military induction and their post recruit exposures to combat violence in their first or second deployments. These prior violence exposures, together with combat exposures, comprise the memories in a fear network the redeployed Marine brings home, synergistically enhancing the risk to develop PTSD, and for some, the clinically relevant disorder. While the PCL-C instrument has proven to be an effective screen for PTSD, care should be taken to include a complete and accurate accounting of traumatic combat exposures and types in the immediate post deployment window, as well as any prior violence exposures when contemplating a clinical diagnosis of PTSD.

## Competing interests

The authors declare that they have no competing interests.

## Authors' contributions

All authors contributed to study concept, design, and drafted the manuscript. CP conceived the study, and directed its design and coordination, and executed statistical analyses. CL prepared the master data file and executed statistical analyses. BS provided oversight of statistical methods. GG designed the table layouts. All authors read and approved the final manuscript.

## Funding

This represents Naval Health Research Center report #08-46, supported by the Department of Defense, under work unit No 60002. Funding sources did not play any other role. The views expressed in this article are those of the authors and do not reflect the official policy or position of the US Department of the Navy; US Department of the Army; US Department of the Air Force; US Marine Corps; or US Department of Defense; Approved for public release. This research has been conducted in compliance with all applicable federal regulations governing the protection of human subjects in research (Protocol NHRC.2003.0027).

## Pre-publication history

The pre-publication history for this paper can be accessed here:

http://www.biomedcentral.com/1471-244X/10/52/prepub

## References

[B1] Association AP Diagnostic and Statistical Manual of Mental Disorders DSM-IV-TR 1994 4 Washington, DC: American Psychiatric Association

[B2] Breslau N Davis GC Andreski P Peterson E Traumatic events and posttraumatic stress disorder in an urban population of young adults Arch Gen Psychiatry 1991 48 3 216 222 1996917 10.1001/archpsyc.1991.01810270028003

[B3] Davidson JR Hughes D Blazer DG George LK Post-traumatic stress disorder in the community: an epidemiological study Psychol Med 1991 21 3 713 721 10.1017/S0033291700022352 1946860

[B4] Helzer JE Robins LN McEvoy L Post-traumatic stress disorder in the general population. Findings of the epidemiologic catchment area survey N Engl J Med 1987 317 26 1630 1634 3683502 10.1056/NEJM198712243172604

[B5] Bremner JD Southwick SM Johnson DR Yehuda R Charney DS Childhood physical abuse and combat-related posttraumatic stress disorder in Vietnam veterans Am J Psychiatry 1993 150 2 235 239 8422073 10.1176/ajp.150.2.235

[B6] Brewin CR Andrews B Valentine JD Meta-analysis of risk factors for posttraumatic stress disorder in trauma-exposed adults J Consult Clin Psychol 2000 68 5 748 766 10.1037/0022-006X.68.5.748 11068961

[B7] Clancy CP Graybeal A Tompson WP Badgett KS Feldman ME Calhoun PS Erkanli A Hertzberg MA Beckham JC Lifetime trauma exposure in veterans with military-related posttraumatic stress disorder: association with current symptomatology J Clin Psychiatry 2006 67 9 1346 1353 10.4088/JCP.v67n0904 17017820

[B8] Foy DW Card JJ Combat-related post-traumatic stress disorder etiology: replicated findings in a national sample of Vietnam-era men J Clin Psychol 1987 43 1 28 31 10.1002/1097-4679(198701)43:1<28::AID-JCLP2270430105>3.0.CO;2-J 3558838

[B9] King DW King LA Foy DW Gudanowski DM Prewar factors in combat-related posttraumatic stress disorder: structural equation modeling with a national sample of female and male Vietnam veterans J Consult Clin Psychol 1996 64 3 520 531 10.1037/0022-006X.64.3.520 8698946

[B10] King DW King LA Foy DW Keane TM Fairbank JA Posttraumatic stress disorder in a national sample of female and male Vietnam veterans: risk factors, war-zone stressors, and resilience-recovery variables J Abnorm Psychol 1999 108 1 164 170 10.1037/0021-843X.108.1.164 10067002

[B11] Koenen KC Stellman JM Stellman SD Sommer JF Jr Risk factors for course of posttraumatic stress disorder among Vietnam veterans: a 14-year follow-up of American Legionnaires J Consult Clin Psychol 2003 71 6 980 986 10.1037/0022-006X.71.6.980 14622073

[B12] Lapp KG Bosworth HB Strauss JL Stechuchak KM Horner RD Calhoun PS Meador KG Lipper S Butterfield MI Lifetime sexual and physical victimization among male veterans with combat-related post-traumatic stress disorder Mil Med 2005 170 9 787 790 16261985 10.7205/milmed.170.9.787

[B13] Smith T Wingard D Ryan M Kritz-Silverstein D Slymen D Sallis J Prior assault increases the likelihood of new-onset PTSD after combat deployment Epidemiology 2008 19 3 505 512 10.1097/EDE.0b013e31816a9dff 18414091

[B14] Smith TC Ryan MA Wingard DL Slymen DJ Sallis JF Kritz-Silverstein D New onset and persistent symptoms of post-traumatic stress disorder self reported after deployment and combat exposures: prospective population based US military cohort study BMJ 2008 336 7640 366 371 10.1136/bmj.39430.638241.AE 18198395 PMC2244768

[B15] Iversen AC Fear NT Ehlers A Hacker Hughes J Hull L Earnshaw M Greenberg N Rona R Wessely S Hotopf M Risk factors for post-traumatic stress disorder among UK Armed Forces personnel Psychol Med 2008 38 4 511 522 10.1017/S0033291708002778 18226287 PMC3785135

[B16] Baker DG Mendenhall CL Simbartl LA Magan LK Steinberg JL Relationship between posttraumatic stress disorder and self-reported physical symptoms in Persian Gulf War veterans Arch Intern Med 1997 157 18 2076 2078 10.1001/archinte.157.18.2076 9382663

[B17] Foy DW Sipprelle RC Rueger DB Carroll EM Etiology of posttraumatic stress disorder in Vietnam veterans: analysis of premilitary, military, and combat exposure influences J Consult Clin Psychol 1984 52 1 79 87 10.1037/0022-006X.52.1.79 6699251

[B18] Kang HK Natelson BH Mahan CM Lee KY Murphy FM Post-traumatic stress disorder and chronic fatigue syndrome-like illness among Gulf War veterans: a population-based survey of 30,000 veterans Am J Epidemiol 2003 157 2 141 148 10.1093/aje/kwf187 12522021

[B19] Roy-Byrne P Arguelles L Vitek ME Goldberg J Keane TM True WR Pitman RK Persistence and change of PTSD symptomatology--a longitudinal co-twin control analysis of the Vietnam Era Twin Registry Soc Psychiatry Psychiatr Epidemiol 2004 39 9 681 685 10.1007/s00127-004-0810-0 15672287

[B20] Vogt DS Tanner LR Risk and resilience factors for posttraumatic stress symptomatology in Gulf War I veterans J Trauma Stress 2007 20 1 27 38 10.1002/jts.20187 17345645

[B21] Hoge CW Castro CA Messer SC McGurk D Cotting DI Koffman RL Combat duty in Iraq and Afghanistan, mental health problems, and barriers to care N Engl J Med 2004 351 1 13 22 10.1056/NEJMoa040603 15229303

[B22] Kolkow TT Spira JL Morse JS Grieger TA Post-traumatic stress disorder and depression in health care providers returning from deployment to Iraq and Afghanistan Mil Med 2007 172 5 451 455 17521088 10.7205/milmed.172.5.451

[B23] Hyams KC Barrett DH Duque D Engel CC Friedl K Gray G Hogan B Kaforski G Murphy F North R The Recruit assessment Program: a program to collect comprehensive baseline health data from U.S. military personnel Mil Med 2002 167 1 44 47 11799812

[B24] Lane SE Young S Bayer L Hogan B Hyams KC Ryan MAK Recruit Assessment Program: Implementation at Marine Corps Recruit Depot, San Diego 2002 02 San Diego: Naval Health Research Center

[B25] Dillman D Mail and telephone surveys: The Total Design Method 1978 xvi New York: John Wiley & Sons

[B26] Weathers FW Litz BT Herman DS Huska JA Keane TM The PTSD Checklist (PCL): reliability, validity, and diagnostic utility Paper presented at the Annual Meeting of International Society for Traumatic Stress Studies: 1993; San Antonio, TX 1993 http://www.pdhealth.mil/library/downloads/PCL_sychometrics.doc

[B27] Blanchard EB Jones-Alexander J Buckley TC Forneris CA Psychometric properties of the PTSD Checklist (PCL) Behav Res Ther 1996 34 8 669 673 10.1016/0005-7967(96)00033-2 8870294

[B28] Hoge CW Castro CA Messer SC McGurk D Cotting DI Koffman RL Combat duty in Iraq and Afghanistan, mental health problems, and barriers to care N Engl J Med 2004 351 1 13 22 10.1056/NEJMoa040603 15229303

[B29] Wright KM Huffman AH Adler AB Castro CA Psychological screening program overview Mil Med 2002 167 10 853 861 12392255

[B30] Brewin CR Systematic review of screening instruments for adults at risk of PTSD J Trauma Stress 2005 18 1 53 62 10.1002/jts.20007 16281196

[B31] Dube SR Anda RF Felitti VJ Chapman DP Williamson DF Giles WH Childhood abuse, household dysfunction, and the risk of attempted suicide throughout the life span: findings from the Adverse Childhood Experiences Study Jama 2001 286 24 3089 3096 10.1001/jama.286.24.3089 11754674

[B32] Felitti VJ Anda RF Nordenberg D Williamson DF Spitz AM Edwards V Koss MP Marks JS Relationship of childhood abuse and household dysfunction to many of the leading causes of death in adults. The Adverse Childhood Experiences (ACE) Study Am J Prev Med 1998 14 4 245 258 10.1016/S0749-3797(98)00017-8 9635069

[B33] Bernstein DP Fink L Handelsman L Foote J Lovejoy M Wenzel K Sapareto E Ruggiero J Initial reliability and validity of a new retrospective measure of child abuse and neglect Am J Psychiatry 1994 151 8 1132 1136 8037246 10.1176/ajp.151.8.1132

[B34] Bernstein DP Stein JA Newcomb MD Walker E Pogge D Ahluvalia T Stokes J Handelsman L Medrano M Desmond D Development and validation of a brief screening version of the Childhood Trauma Questionnaire Child Abuse Negl 2003 27 2 169 190 10.1016/S0145-2134(02)00541-0 12615092

[B35] Straus M GR J Physical Violence in American Families: Risk Factors and Adaptations to Violence in 8,145 Families 1990 New Brunswick, NJ: Transaction Press

[B36] Grieger TA Cozza SJ Ursano RJ Hoge C Martinez PE Engel CC Wain HJ Posttraumatic stress disorder and depression in battle-injured soldiers Am J Psychiatry 2006 163 10 1777 1783 quiz 1860 10.1176/appi.ajp.163.10.1777 17012689

[B37] Friedman MJ Schnurr PP McDonagh-Coyle A Post-traumatic stress disorder in the military veteran Psychiatr Clin North Am 1994 17 2 265 277 7937358

[B38] Breslau N Davis GC Andreski P Risk factors for PTSD-related traumatic events: a prospective analysis Am J Psychiatry 1995 152 4 529 535 7694900 10.1176/ajp.152.4.529

[B39] Ozer EJ Best SR Lipsey TL Weiss DS Predictors of posttraumatic stress disorder and symptoms in adults: a meta-analysis Psychol Bull 2003 129 1 52 73 10.1037/0033-2909.129.1.52 12555794

[B40] Neuner F Schauer M Karunakara U Klaschik C Robert C Elbert T Psychological trauma and evidence for enhanced vulnerability for posttraumatic stress disorder through previous trauma among West Nile refugees BMC Psychiatry 2004 4 34 10.1186/1471-244X-4-34 15504233 PMC529265

[B41] Schauer M Neuner F Karunakara U Klaschik C Robert C Elbert T PTSD and the "building block" effect of psychological trauma among West Nile Africans ESTSS (European Society for Traumatic Stress Studies) Bulletin 2003 10 2 5 6

[B42] Kolassa I-T Ertl V Eckart C Gloeckner F Kolassa S A P The probability of spontaneous remission from PTSD depends on the number of traumatic event types experienced Psych Trauma: Theory Res, And Pract in press

[B43] Cohen S Wills TA Stress, social support, and the buffering hypothesis Psychol Bull 1985 98 2 310 357 10.1037/0033-2909.98.2.310 3901065

[B44] Holahan CJ Moos RH Social support and psychological distress: a longitudinal analysis J Abnorm Psychol 1981 90 4 365 370 10.1037/0021-843X.90.4.365 7264067

[B45] King LA King DW Fairbank JA Keane TM Adams GA Resilience-recovery factors in post-traumatic stress disorder among female and male Vietnam veterans: hardiness, postwar social support, and additional stressful life events J Pers Soc Psychol 1998 74 2 420 434 10.1037/0022-3514.74.2.420 9491585

[B46] Taft CT Stern AS King LA King DW Modeling physical health and functional health status: the role of combat exposure, posttraumatic stress disorder, and personal resource attributes J Trauma Stress 1999 12 1 3 23 10.1023/A:1024786030358 10027139

[B47] Schnurr PP Lunney CA Sengupta A Risk factors for the development versus maintenance of posttraumatic stress disorder J Trauma Stress 2004 17 2 85 95 10.1023/B:JOTS.0000022614.21794.f4 15141781

[B48] Benotsch EG Brailey K Vasterling JJ Uddo M Constans JI Sutker PB War zone stress, personal and environmental resources, and PTSD symptoms in Gulf War veterans: a longitudinal perspective J Abnorm Psychol 2000 109 2 205 213 10.1037/0021-843X.109.2.205 10895558

[B49] Brailey K Vasterling JJ Proctor SP Constans JI Friedman MJ PTSD symptoms, life events, and unit cohesion in U.S. soldiers: baseline findings from the neurocognition deployment health study J Trauma Stress 2007 20 4 495 503 10.1002/jts.20234 17721953

[B50] Hammen C Mayol A deMayo R Marks T Initial symptom levels and the life-eventdepression relationship J Abnorm Psychol 1986 95 2 114 122 10.1037/0021-843X.95.2.114 3711434

[B51] Holahan CJ Moos RH Risk, resistance, and psychological distress: a longitudinal analysis with adults and children J Abnorm Psychol 1987 96 1 3 13 10.1037/0021-843X.96.1.3 3558946

[B52] Monroe SM Life events and disorder: event-symptom associations and the course of disorder J Abnorm Psychol 1982 91 1 14 24 10.1037/0021-843X.91.1.14 7056938

[B53] Iversen AC Fear NT Ehlers A Hughes JH Hull L Earnshaw M Greenberg N Rona R Wessely S Hotopf M Risk factors for post-traumatic stress disorder among UK Armed Forces personnel Psychol Med 2008 38 4 511 522 10.1017/S0033291708002778 18226287 PMC3785135

[B54] Smith TC Smith B Jacobson IG Corbeil TE Ryan MA Reliability of standard health assessment instruments in a large, population-based cohort study Ann Epidemiol 2007 17 7 525 532 10.1016/j.annepidem.2006.12.002 17433714

[B55] Hoge CW Auchterlonie JL Milliken CS Mental health problems, use of mental health services, and attrition from military service after returning from deployment to Iraq or Afghanistan JAMA 2006 295 9 1023 1032 10.1001/jama.295.9.1023 16507803

